# Analysis of *Culex* and *Aedes* mosquitoes in southwestern Nigeria revealed no West Nile virus activity

**DOI:** 10.11604/pamj.2016.23.116.7249

**Published:** 2016-03-17

**Authors:** Waidi Folorunso Sule, Daniel Oladimeji Oluwayelu

**Affiliations:** 1 Department of Biological Sciences, Faculty of Basic and Applied Sciences, Osun State University, PMB 4494, Oke-Baale, Osogbo, 230212, Osun State, Nigeria; 2 Department of Veterinary Microbiology and Parasitology, Faculty of Veterinary Medicine, University of Ibadan, Ibadan, Nigeria

**Keywords:** Mosquitoes, WNV-RNA, real-time RT-PCR, infection risk, southwestern Nigeria

## Abstract

**Introduction:**

Amplification and transmission of West Nile virus (WNV) by mosquitoes are driven by presence and number of viraemic/susceptible avian hosts.

**Methods:**

In order to predict risk of WNV infection to humans, we collected mosquitoes from horse stables in Lagos and Ibadan, southwestern Nigeria. The mosquitoes were sorted and tested in pools with real-time RT-PCR to detect WNV (or flavivirus) RNA using WNV-specific primers and probes, as well as, pan-flavivirus-specific primers in two-step real-time RT-PCR. Minimum infection rate (MIR) was used to estimate mosquito infection rate.

**Results:**

Only two genera of mosquitoes were caught (*Culex*, 98.9% and *Aedes*, 1.0%) totalling 4,112 females. None of the 424 mosquito pools tested was positive for WNV RNA; consequently the MIR was zero. Sequencing and BLAST analysis of amplicons detected in pan-flavivirus primer-mediated RT-PCR gave a consensus sequence of 28S rRNA of *Culex quinquefasciatus* suggesting integration of flaviviral RNA into mosquito genome.

**Conclusion:**

While the latter finding requires further investigation, we conclude there was little or no risk of human infection with WNV in the study areas during sampling. There was predominance of *Culex* mosquito, a competent WNV vector, around horse stables in the study areas. However, mosquito surveillance needs to continue for prompt detection of WNV activity in mosquitoes.

## Introduction

West Nile Virus (WNV) is a mosquito-borne viral pathogen that belongs to the *Flaviviridae* family. It is closely related to other human pathogens such as yellow fever (YF), dengue (DEN), tickborne encephalitis (TBE), Japanese encephalitis (JE), and Murray Valley encephalitis (MVE) viruses [1]. WNV contains a singlestranded, positive-sense RNA genome of about 11kb that is held in a nucleocapsid. The viral genome is translated as a single polyprotein, which is cleaved into three structural and seven nonstructural (NS) proteins [2]. The WN virion of about 45-50 nm in diameter is contained in a host-derived membrane; the membrane has two viral glycoproteins, the membrane (M) and envelope (E) proteins, embedded in it [3].

WNV mainly infects birds but can infect many other species including humans [4]. Transmission, mainly via *Culex (Cx)* mosquitoes, occurs by acquisition of the virus by female mosquitoes through blood meal from infected birds (amplifying reservoir hosts) and introduction of the infectious virions to other susceptible birds and mammals during subsequent blood meals [5]. Unlike humans, horses and other mammals that are reproductive dead-end hosts [6], birds have high and durable viral titer that allows them transmit the virus to biting mosquitoes. Besides *Culex spp*, other mosquitoes that efficiently transmit WNV are *Aedes (Ae) albopictus*, *Ae vexans* and *Ochlerotatus triseriatus* [7].

WNV has become a global threat of public health and veterinary significance [8]. Thus, there is a need to conduct surveillance for the virus in mosquitoes in order to correctly provide spatio-temporal information on risk of infection in humans (and other vertebrates) [9]. WNV surveillance consists of two distinct but complementary activities: the epidemiological and environmental surveillance activities. The latter involves monitoring of local WNV activity in vectors and non-human vertebrate hosts in advance of epidemic activity that affects humans [10, 11]. WNV activity can be monitored by testing adult mosquitoes for virus infection [12] through molecular detection of viral RNA using real-time RT-PCR. This method is preferred not only for its sensitivity (detects about 0.1 PFU/ml), but also for its short turn-around time of about 4 hours [11, 13]. According to Condotta *et al*. [14], infection rate, which is the proportion of mosquitoes in the environment infected with virus, can be estimated using either minimum infection rate (MIR) or maximum likelihood estimate (MLE). The MIR is the number of mosquito pools infected per 1000 female mosquitoes tested and is most appropriate when < 1000 female mosquitoes are tested. Consequently, MIR of zero suggests no viral activity in study mosquitoes which implies little or no risk of human infection, MIR between 0.1 and 3.9 implies presence of some viral activity which necessitates increased vigilance and mosquito testing, while MIR of 4.0 or above indicates presence of high level of viral activity in the area and that human infections are imminent, if not already present. Compared to testing individual mosquitoes, testing sets of pooled mosquitoes of the same species is logistically the easiest and most cost-effective approach for WNV testing [14].

In Nigeria, there is no recent report of WNV isolation from mosquitoes though its RNA has been reportedly detected in mosquitoes caught in the northeastern and southwestern parts of the country [15]. In addition, considering that WNV is an RNA virus with higher propensity for mutational changes *vis-à-vis* climatic/environmental changes and global travels, there is a need to test mosquitoes in the rainforest southwestern Nigeria for WNV activity and viral properties with the view to predicting risk of WNV infection to humans and instituting necessary control response.

## Methods

### Study area

**Figure 1:**
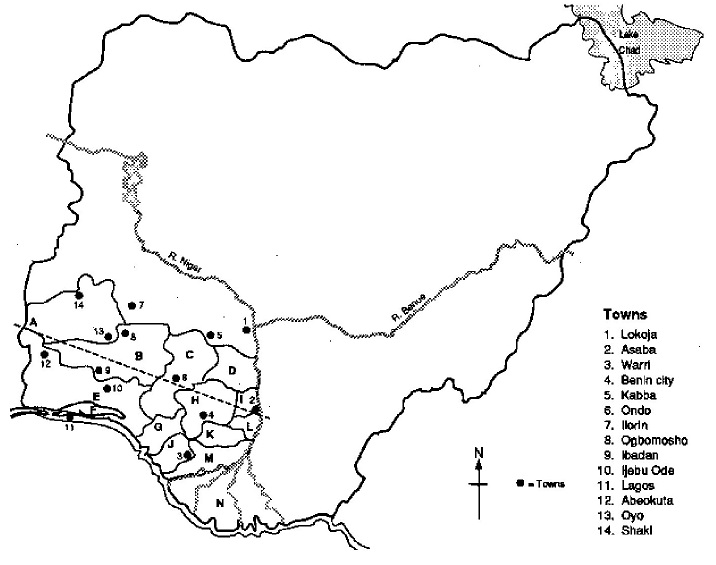
Map of Nigeria showing southwestern states, inside which are the locations (9 as Ibadan and 11 as Lagos) of mosquito sampling (www.fao.org_wairdocs_ilri_x5458e_x5458e0a.gif)

Mosquitoes were collected between June, 2013 and January, 2014 from Onosa (N06.47001°, E003.80226°) and Ajah (N06.46700°, E003.57255°) horse ranches in Lagos State, and from Eleyele Polo club (N07.4036°, E003.8726°) in Ibadan, Oyo State, both in southwestern Nigeria (Figure 1). Lagos State, with a coastline of approximately 180 km, has coastal wetlands and upland rainforest as dominant ecozones. The vegetation cover is mostly a mosaic of mangrove swamps, freshwater swamps, secondary forest, farmland and fallow land. The soils are mostly deep and poorly drained. Its climate is wet equatorial influenced by its nearness to the equator and the Gulf of Guinea. It enjoys rainy season with two peaks: May to July and September to October, with the former being the heaviest. Floods characterize the peaks due to the poor surface drainage systems of the coastal lowlands. The mean annual rainfall ranges from 1,567.2mm in the north-western part of the state to 1,750mm in the mainland areas. The temperature of the state is generally consistently high, with the mean monthly maximum temperature of about 30°C [16, 17]. The climate of Oyo State on the other hand, is typically West African Monsoon marked by distinct seasonal shifts in wind patterns. The rainy season that starts in Oyo state during the first week of March with storms averages 8 months in a year with average annual rainfall being over 1,000mm while the dry season occurs from November to February when dry, dust-laden winds blow from the Sahara desert heralding the harmattan period. The vegetation is rain forest and derived savannah, and average temperature is between 18.9°C to 35°C [18, 19]. Most of the periods of mosquito collection coincided with the wet seasons in the two states.

### Study design, mosquito collection and storage

This is a mosquito surveillance study and the mosquitoes were collected using two different traps - BioGents sentinel trap (Biogents, Regensburg, Germany) and the Centers for Disease Control and Prevention (CDC) light mosquito trap (John W. Hock Company, Gainesville, FL, USA) [14, 20]. The traps are herein referred to as “BG” and “CDC” traps. The 12V battery-powered CDC and BG mosquito traps were set at the entrance of and around the horse boxes in Eleyele polo club, and at Ajah and Onosa stables. The two traps were used in order to catch as many mosquitoes as possible and to have representative catch of the mosquitoes prevalent at each location. The CDC light trap attracts most flying insects while the BG trap, equipped with Biogents Sweetscent™ attractant that simulates human body odour, mostly attracts bloodseeking mosquitoes. The traps were set from dusk (about 1800 hours) to dawn (about 0700 hours) [21]. Trapped mosquitoes in the receptacles were anaesthetized on ice packs; when they had become very weak or dead, they were packed into cryovials and transported on ice to the Virology laboratory, Department of Veterinary Microbiology and Parasitology, Faculty of Veterinary Medicine, University of Ibadan, Ibadan where they were stored at -80°C until analyzed.

### Mosquito sorting, homogenization and RNA extraction

With the assistance of an entomologist and using taxonomic keys [22], the mosquitoes were sorted on ice under a stereo-microscope into pools based on location of collection, type of mosquito trap, date of collection, genus and gender. A pool contained between 1 and 12 (average of 10) adult mosquitoes of same genus. Only the females were further analyzed for presence of WNV. Each pool of female mosquitoes was homogenized using the QIAquick^®^ PCR Purification Kit (QIAGEN, Germany) according to the manufacturer's instructions. Briefly, two 5mm stainless steel beads were added to each vial of mosquitoes followed by addition of 600µl of lysis buffer (buffer RLT) which contains guanidine thiocyanate. The vials were tightly covered with their snap-caps and loaded into TissueLyser^®^ (QIAGEN, Germany) already set to vibrate at 25 beats per second for 2 minutes. The homogenates were then transferred to a refrigerated centrifuge and spun at 13,200 rpm for 3 minutes. Without touching any pellet, the supernatant from each tube was dispensed into correspondingly labeled new 1.5ml tube and stored at -80°C until used for RNA extraction. RNA was extracted from each 600µl mosquito lysate using RNeasy^®^ Mini Kit (QIAGEN, Germany) spin column according to manufacturer's instructions. A volume of 50µl RNase-free water was used to elute the sample RNA into 1.5ml collection tube which was stored at -80°C until further analyzed.

### RNA testing using One- and Two-step real-time RT-PCR

One-step real-time RT-PCR to detect WNV RNA was performed on the RNA samples with iScriptTM One-Step RT-PCR Kit (BIORAD, USA) as previously described [23]. Pipetting and plate preparation were done on ice crystals thus: 2µl RNA template was added to 48µl master mix containing 1µl iScript reverse transcriptase, 1µl each of forward and reverse primers (5pmol/µl each), 1µl WNV Linke probe (2.5pmol/µl) [24], 25µl of 2X RT-PCR reaction buffer for probes and 19µl nuclease-free water. HPLC grade water was used as no-template control (NTC) while RNA templates of WNV lineage 1 (ArB3573/82), WNV-goose Israel 1998 and WNV Lineage 2-Madagascar were included in different reactions as WNV positive controls. The sequences of primers and probes used are shown in Table 1. The reaction tubes were incubated in real-time PCR system (STRATAGENE^®^ MX 3000P, Agilent Technologies, USA) which was programmed as follows: 50°C for 10 minutes for reverse transcription, 95°C for 5 minutes to inactivate reverse transcriptase and activate Taq polymerase, then 45 cycles of 95°C for 15 seconds and 55°C for 30 seconds for cDNA amplification. The machine was programmed to collect data for FAM. Following the One-step real-time RT-PCR using WNV-specific primers, we tested some RNA samples in two-step RT-PCR, using pan-flavivirus- and pan-alphavirus-specific primers with RNAs of WNV and louping ill virus as positive controls for pan-flavivirus reaction and those of Chikungunya and Getah viruses as positive controls for the pan-alphavirus reaction. The pan-flavivirus and pan-alphavirus primers are shown in Table 2.

**Table 1:** Genomic position and nucleotide sequences of primers and probe for real-time RT-PCR [24]

Primer	Genomic position	sequence	Product size
WNV Linke forward	10-33	5’-CCTgTgTgAgCTgACAAACTTAgT-3’	144bp
WNV Linke reverse	132-153	5’-gCgTTTTAgCATATTgACAgCC-3’	
WNV Linke probe	89-113	5’-[6FAM] CCTggTTTCTTAgACATCgAgATCTXCgTgCp[TAMRA]-3’	

**Table 2:** Genomic positions and nucleotide sequences of primers for pan-flavivirus and pan-alphavirus real-time PCR [23, 25]

Primer	Genomic position	Sequence	Product size
Pan-flavivirus forward	9103–9120	5’-GCMATHTGGTWCATGTGG-3’	200 bp
Pan-flavivirus reverse	9283–9305	5’-GTRTCCCAKCCDGCNGTRTC-3’	
Pan-alphavirus forward	6971-6997	5’-TGGCGCTATGATGAAATCTGGAATGTT-3’	214 bp
Pan-alphavirus reverse	7086-7109	5’-TACGATGTTGTCGTCGCCGATGAA -3’	

For the two-step reaction, 2µl RNA template was added to 18µl master mix containing 4µl RT buffer (5X), 1µl dNTPs (10mM), 1µl DTT (0.1M), 2µl Random hexamers (50ng/µl) (Roche^®^), 1µl RNasin (40U/µl), 1µl MMLV-RT (200U/µl) and 8µl nuclease-free water, and incubated on heating block at 42°C for 1 hour. Thirty microliter of HPLC grade water used as NTC was added to each cooled cDNA sample to dilute it. From the latter, 5µl each was taken and added as template respectively to 35µl pan-flavivirus master mix containing 1µl Flavi-F (10pmol/µl), 1µl Flavi-R (10pmol/µl), 20µl SYBR^®^ Green JumpStart^®^ Taq ReadyMix^®^, and 13µl nuclease-free water, and to 35µl pan-alphavirus master mix containing 1µl VIR 2052F Alpha (10pmol/µl), 1µl VIR 2052R Alpha (10pmol/µl), 20µl SYBR^®^ Green JumpStart^®^ Taq ReadyMix^®^, and 13µl nuclease-free water in different reaction tubes. The cycling conditions were 95°C for 10 minutes, and 50 cycles of 95°C for 30s, 55°C for 30s and 72°C for 30s, followed by 1 cycle of 95°C for 60s, 55°C for 30s and 95°C for 30s (the machine was set to process dissociation curve starting at 72°C for 10s).

**Gel electrophoresis:** a 2µl aliquot of each PCR product was examined by electrophoresis on 1.8% agarose gel containing 5µl of SYBR^®^ safe (Invitrogen, USA), PhiX174 phage DNA was used as molecular weight marker and the gel was visualized with GelDoc XR Scanner (BIORAD, USA).

**Sequencing of PCR products:** the amplicon from each RNA sample that gave detectable band in agarose gel electrophoresis was cleaned up using the QIAquick PCR Purification kit (QIAGEN, Germany) according to manufacturer's protocol. The purified PCR products were subjected to nucleotide sequencing using Flavi-F: GCMATHTGGTWCATGTGG and Flavi-R: GTRTCCCAKCCDGCNGTRTC primers with ABI PRISM (r) 3100 Genetic Analyzer (Applied Biosystems Inc.).

**Estimation of mosquito minimum infection rate (MIR):** mosquito minimum infection rate (MIR) was estimated using the formula: (number of WNV-positive mosquito pools/total number of mosquitoes tested) x 1000 [21].

**Data analysis:** the results obtained were presented with descriptive statistics while nucleotide sequences generated were analyzed by performing BLAST search in the GenBank (NCBI).

## Results

**Figure 2:**
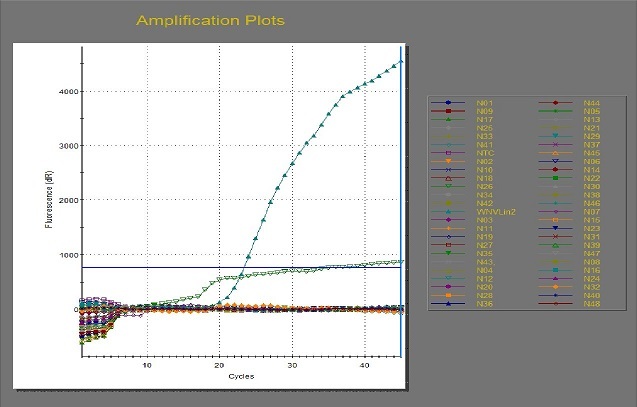
Real-time amplification of 48 RNA samples using WNV Linke primers and probe

**Figure 3:**
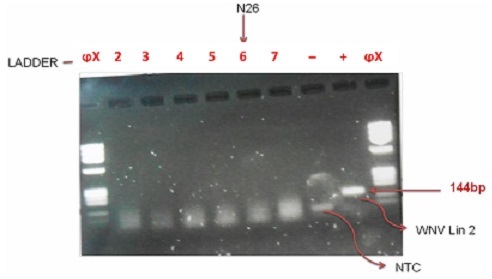
RT-PCR detection of 6 RNA samples from 6 pools of female mosquitoes. Lanes 1and 10: Molecular weight markers, Lanes 2 – 7: Test samples, Lane 8: No template control, Lane 9: positive control

In all, 4,112 female mosquitoes belonging to two genera, *Culex* and *Aedes*, were identified (Table 3). They were sorted into 424 pools comprising 413 pools of *Culex* (n = 4,070; 98.9%) and 11 pools of *Aedes* (n = 42; 1.0%). No male *Aedes* was trapped. Real-time RT-PCR of the first batch of 48 RNA samples did not give any cycle threshold (Ct) value (i.e. no detectable WNV RNA), except for a sample (N26) that gave a weird amplification plot as shown in Figure 2. The sample was a pool of 10 female *Culex* mosquitoes from Onosa horse stable. However, the sample (Figure 3, Lane 6) did not yield the desired band following gel electrophoresis.

**Table 3:** Distribution of mosquito samples collected in southwestern Nigeria

Location	Overall pool (n)	Genus Pool (n)				Trap Pool (n)		Year Pool (n)	
		*Aedes*		*Culex*		CDC	BG	2013	2014
		Male	Female	Male	Female				
**Onosa**	383 (3784)	-	6 (30)	-	377(3754)	333 (3296)	50 (488)	383(3784)	-
**Ajah**	36 (321)	-	3 (10)	1 (2)	32 (309)	29 (264)	7 (57)	36 (321)	-
**Ibadan**	8 (11)	-	2 (2)	2 (2)	4 (7)	4 (4)	4 (7)	-	8 (11)
**Total**	427 (4116)	-	11(42)	3 (4)	413(4070)	366(3564)	61 (552)	419 (4105)	8 (11)

**Figure 4:**
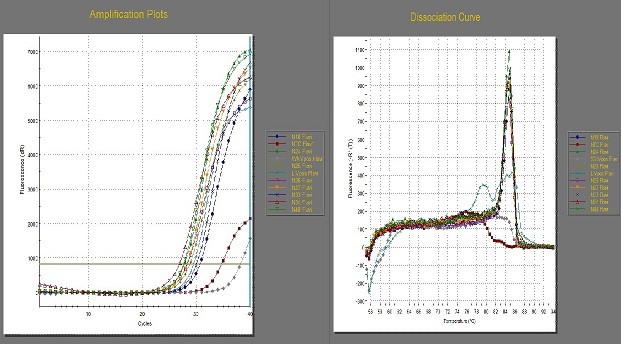
Two-step RT-qPCR amplification plots of 8 RNA samples using pan-flavivirus primers and the corresponding dissociation curves

**Figure 5:**
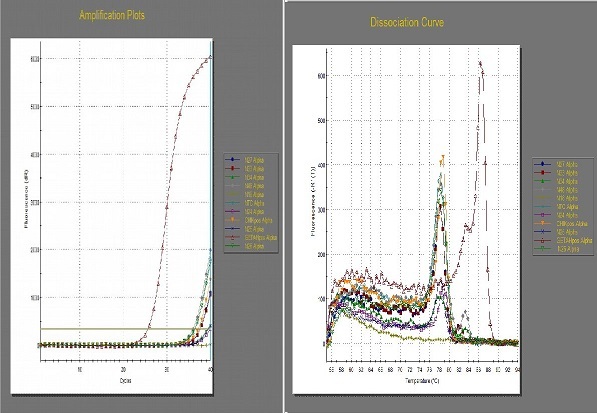
Two-step RT-qPCR amplification plots of the 8 RNA samples using pan-alphavirus primers and their dissociation curves

**Figure 6:**
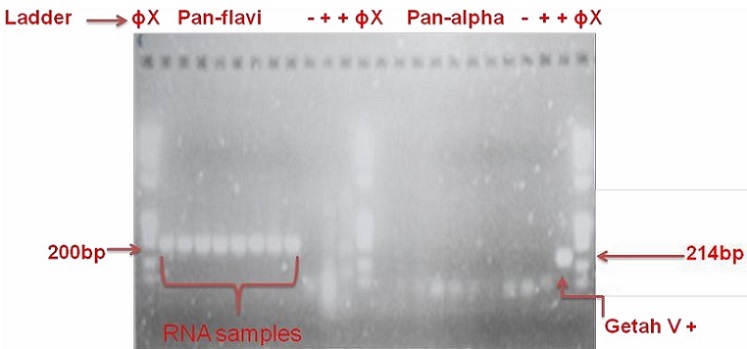
Gel detection of the 8 RNA samples from two-step RT-qPCR of pan-flavivirus and pan-alphavirus reactions: lanes 1, 13 and 25: Molecular weight markers, lanes 2 – 9: Test samples, lanes 10 and 22: No template controls, lanes 11, 12, 23 and 24: positive controls, lanes 14-21: test samples

The pan-flavivirus and pan-alphavirus primer-mediated two-step RT real-time PCR of sample N26 and seven others selected at random around it gave amplification plots and dissociation curves as shown in Figure 4; the pan-alphavirus reaction, however, was negative (Figure 5). Figure 6 shows the gel electrophoresis result of the samples; the pan-flavivirus reactions showed positive bands, while those of pan-alphavirus gave negative bands. The remaining 37 RNA samples also gave *Ct* values in pan-flavivirus reaction (data not shown).

Sequencing of the 8 samples that gave detectable RNA from the pan-flavivirus reaction (Figure 6) revealed a consensus sequence of 191 nucleotides as shown below: 5’-AAGTTGCAATATGGTACATGTGGTGATATTTAGCTTTAGAAGGAGTTTACCTCCCACTTTGTGCTGCACTATCAAGCAACA

CGACTCCATGGAAAAATTTTCCACCATCAGCACCGTCCTACGGGCCTATCACCCTCTATGGGAGTAAAAGCCACATTCTAGT

TGAACTTGGACACCGCCGGCTGGGACAC -3’.

BLAST analysis of the sequence in GenBank revealed it was identical to the 28S ribosomal RNA (rRNA) of host mosquitoes with the sequence of *Culex quinquefasciatus* producing significant homology. When the remaining samples were subjected to WNV primer mediated - real-time RT-PCR, only the positive controls gave Ct values while the samples were all negative (data not shown).

**Minimum mosquito infection rate (MIR):** estimation of MIR gave overall value of zero; the genus-, location- and trap type-specific MIRs were, of course, also zero.

## Discussion

This surveillance study was conducted to investigate WNV infection in mosquitoes in the rainforest ecological zone of south western Nigeria. It was noticeable that greater proportion of *Culex* mosquitoes - the maintenance/bridge vector in enzootic or epidemic cycle of WNV - than Aedes were collected. A probable reason for this is that the former are usually more active from dusk to dawn (night-biters) unlike *Aedes* that are mostly day-biting [26]. Baba *et al*. [15], LaBeaud *et al*. [21], Özer *et al*. [27] and Vaux et al. [28] also collected greater proportion of *Culex* mosquitoes in their studies.

Real-time RT-PCR for detection of WNV in mosquitoes is very sensitive and specific with short turn-around time; hence it is widely used for arboviral surveillance [8, 11]. We processed study mosquitoes according to established protocols and manufacturer's guide and observed that the first batch of 48 RNA samples did not give any Ct values indicating presence of WNV RNA in the mosquitoes except a weird amplification plot of sample N26 which gave a Ct value of 34.93, unlike the positive WNV control with 23.39 Ct value (Figure 2). The remaining RNA samples had detectable RNA in pan-flavivirus RT-PCR but sequence analysis showed the consensus sequence as 28S rRNA of *Culex quinquefasciatus*. A possible explanation for this is integration of flaviviral RNA (WNV RNA inclusive) into genome of host *Culex* mosquitoes. Integration of flaviviral RNA (though not specifically for WNV) into host mosquito's genome have been reported [29, 30]; we were, however, not sure whether or not this was the case in our own study. A future study is recommended to further elucidate on this.

The real-time-RT-PCR of the remaining samples did not detect any WNV RNA. Testing of mosquitoes (*Culex* and *Aedes* spp) using real-time or conventional RT-PCR without detecting WNV (or arboviral) RNA has been previously reported [8, 20, 27, 28]. The fact that WNV RNA was not detected in any of the mosquito pools tested in this study is worth noting considering that serologic studies on horse [31] and human sera (unpublished data) and previous serologic studies in humans [15, 32] showed high prevalence of anti-WNV antibodies in Nigeria. However, high anti-WNV antibody prevalence/herd immunity among amplifying host birds inversely correlated with mosquito infection rate [33, 34]. These observations might explain why, in spite of reports of WNV-specific antibodies in humans and horses, none of the tested female mosquitoes yielded WNV RNA. They might also be a possible reason for the absence of reports of WN disease outbreak or WNV-induced encephalitis in humans or horses in Nigeria.

Though we did not test birds in the study locations, the established close association between amplifying host birds and biting mosquitoes suggest that as at the time of sampling, the collected mosquitoes did not pick WNV from the local amplifying host birds during blood feeding. This could be due to absence of WN viremia or low titer viraemia caused by high prevalence of anti-WNV antibodies in the local amplifying host birds or very low number of such birds. Additionally, the caught mosquitoes were less likely to pick WNV from the sampled horses due to high antibody prevalence and presence of neutralizing antibodies [31] which have been reported to make viremia in horses or humans transient [35]. It is also possible that infectious female mosquitoes had died out, due to their short life-span (about 2 weeks for female mosquitoes), before the sampling period. Moreover, the mosquito infection rate of zero obtained in this study implies that the study mosquitoes were apparently uninfected with WNV or, there was possibility of silent undetectable WNV or other arboviral transmission cycle as noted by Roiz *et al*[20].

## Conclusion

As at the time of this study, there was no detectable (active) WNV infection of mosquitoes in the study areas, at least, not at a level that could precipitate WN disease. The dominance of *Culex* mosquitoes, however, indicate establishment of maintenance/bridge vector of WNV in southwestern Nigeria. We suggest mosquito sampling around breeding sites of birds (resident/migratory, aquatic/terrestrial) which may yield detectable WNV RNA as well as screening of birds around the same horse stables for presence of WNV activity. Also, mosquito surveillance needs to continue for prompt detection of WNV activity in these mosquitoes.

### What is known about this topic

- Mosquitoes - *Cx. quinquefasciatus* and *mansonia sp*. - were collected from northeastern and south western Nigeria by some workers with the aim of detecting WNV in them. In their study and others conducted outside Nigeria, *Cx. quinquefasciatus* mosquitoes, globally recognized as competent vectors of WNV, were the predominant species identified.
- While few entomologic surveillance studies for WNV and other arboviruses detected viral RNA, many of such studies reported no detection of WNV RNA.
- Some entomologic studies for WNV have reported integration of flaviviral RNA into chromosomes of *Aedes*host mosquitoes.

### What this study adds

- This current study employed a more sensitive and specific molecular technique – the real-time RT-PCR with additional use of pan-flavivrus- and pan-alphavirus-specific primers. Also, contrary to previous study which was concluded at the gel electrophoresis step, in this study the detected amplicons from the flavivirus real-time RT-PCR were further sequenced and analyzed by BLAST search. This revealed, most likely for the first time in Nigeria, that flaviviral RNA was integrated into the host *Cx. quinquefasciatus* genome, unlike in *Aedes* sp. as previously reported. While this has considerable implication on the evolution of both the WNV and the host vector, it shows by molecular identification of 28S *rRNA*, that the more abundant mosquitoes were actually *Cx. quinquefasciatus*.
- Based on a MIR of zero, this study revealed that there was little or no risk of WNV infection to horses and humans in the study area as at the time of sampling. This highlights the need for continual entomologic surveillance to detect the period of intense WNV transmission (i.e. high risk period) in the study area.
- In line with the last observation, this study further shows the need to conduct surveillance prior to deployment of resources in order to know where and when to direct prevention or control measures/efforts.
